# The mutated tegument protein UL7 attenuates the virulence of herpes simplex virus 1 by reducing the modulation of α-4 gene transcription

**DOI:** 10.1186/s12985-016-0600-9

**Published:** 2016-09-13

**Authors:** Xingli Xu, Shengtao Fan, Jienan Zhou, Ying Zhang, Yanchun Che, Hongzhi Cai, Lichun Wang, Lei Guo, Longding Liu, Qihan Li

**Affiliations:** Institute of Medical Biology, Chinese Academy of Medical Sciences and Peking Union Medical College, Yunnan Key Laboratory of Vaccine Research and Development of Severe Infectious Disease, Kunming, Yunnan China

**Keywords:** Herpes Simplex Virus type I, UL7, α-4 gene, Transcription

## Abstract

**Background:**

UL7, a tegument protein of Herpes Simplex Virus type I (HSV-1), is highly conserved in viral infection and proliferation and has an unknown mechanism of action.

**Methods:**

A HSV-1 UL7 mutant (UL7-MU) was constructed using the CRISPR-cas9 system. The replication rate and plaque morphology were used to analyze the biological characteristics of the wild-type (WT), UL7-MU and MU-complemented P1 viruses. The virulence of the viruses was evaluated in mice. Real-time RT-qPCR and ChIP assays were used to determine the expression levels of relevant genes.

**Results:**

The replication capacity of a recombinant virus (UL7-MU strain) was 10-fold lower than that of the WT strain. The neurovirulence and pathologic effect of the UL7-MU strain were attenuated in infected mice compared with the WT strain. In the latency model, the expression of latency-associated transcript (LAT) in the central nervous system (CNS) and trigeminal nerve was lower in UL7-MU-infected mice than in WT strain-infected mice. The transcription level of the immediate-early gene α-4 in UL7-MU-infected cells was reduced by approximately 2-fold compared with the clear transcriptional peak identified in WT strain-infected Vero cells within 7 h post-infection (p.i.).

**Conclusion:**

By modulating the transcription of the α-4 gene, UL7 may be involved in transcriptional regulation through its interaction with the transcript complex structure of the viral genome during HSV-1 infection.

**Electronic supplementary material:**

The online version of this article (doi:10.1186/s12985-016-0600-9) contains supplementary material, which is available to authorized users.

## Background

Herpes Simplex Virus type I (HSV-1) infection causes a viral disease with an essential effect on human health [1, 2]; the infection is primarily characterized by severe vesicle eruptions around the mouth and lip, with the potential to develop encephalitis with remarkable sequelae [3]. The incidence of herpes in the genital organs has also gradually increased in recent years [4]. Furthermore, once HSV-1 infects the corneal epithelium, the virus then penetrates the nerve terminals and establishes long-term latent infection in the neurological system [5]. Reactivation of latent infections could also induce the clinical manifestations of acute disease development and the associated long-term pathologic processes, which have been strongly linked to the complicated gene structures of HSV-1 and the molecular mechanisms of transcriptional regulation during viral replication [6–8]. As shown in previous studies of viral infection, some HSV-1 genome-encoded proteins, including the critical regulatory factors VP16, VP22 and ICP0, which are involved in the transcriptional modulation of viral genes, are tegument proteins that serve as components of the viral structure and also play important roles in gene replication and virion assembly. Previous studies have demonstrated that during the initiation of immediate early-early-late genes in the HSV-1 genome, HSV-1 uses specific regulatory molecules, which function as the tegument proteins in the completion of viral proliferation in cells, to modulate its complicated transcriptional replication system [9–14]. Exploring viral tegument proteins with unknown functions is essential for understanding the mechanisms of HSV-1 gene transcriptional regulation and for characterizing viral proliferation. In recent years, proteomic analyses have shown that the protein encoded by the UL7 gene comprises approximately 1 % of the total HSV-1 tegument proteins, which has also been confirmed in variety of herpes virus members, such as herpes simplex virus type 2 (HSV-2) and human cytomegalovirus (HCMV), emphasizing the need to characterize the function of the UL7 protein as a regulatory molecule in HSV-1 infection and proliferation [15–19]. A complex produced by the interaction between the UL7 protein and another tegument protein, UL51, has been suggested to directly mediate the entry of UL7 into the cell nucleus upon virion assembly. The UL7-UL51 complex is therefore potentially significant in virion assembly; however, the interaction between UL7 and UL51 has not been shown to be involved in viral infection in vivo [20]. In the current study, the biological function of the UL7 protein during HSV-1 infection is investigated using a mutant strain in which the UL7 gene is modified by the CRISPR/Cas9 system. The UL7 protein exhibited a specific modulatory function by regulating the transcription of the HSV-1 α-4 gene in HSV-1-infected cells.

## Methods

### Cells and viruses

Human embryonic kidney (HEK) 293 T cells and African green monkey kidney Vero cells (ATCC, Manassas, VA, U.S.A.) were maintained in high-glucose Dulbecco’s modified Eagle medium (DMEM, Corning, New York, U.S.A.) supplemented with 10 % fetal bovine serum (FBS). The culture medium was changed to DMEM supplemented with 2 % FBS after viral infection of 293 T and Vero cells. HSV-1 strains 8 F (originated from a clone of F strain) [21] was cultured in 293 T cells in mutant construction assay. HSV-1 8 F and UL7-MU were cultured in Vero cells in proliferation, plaque, quantitative RT-PCR and CHIP assay. The viruses were titered on Vero cells.

### CRISPR/Cas9 g-RNA design of the HSV-1 UL7 gene

Two sets of guide RNAs (g-RNAs) were designed to target the coding sequence of the UL7 gene according to the protocol reported by Ran et al. [22]. The g-RNA sequences were named UL7-1-top, UL7-1-bottom, UL7-2-top and UL7-2-bottom. The genomic region surrounding the CRISPR target site of the UL7 gene was PCR-amplified with the primers UL7-sense and UL7-antisense. The specific primer sets used are listed in Additional file 1: Table S1.

### Plasmid construction and transfection

The g-RNA expression plasmids were constructed according to the protocol reported by Ran et al. Briefly, the top and bottom strands of the oligos for each g-RNA design were resuspended. The dsDNA fragments were annealed and inserted into the Bbs1 (New England BioLabs, Ipswich, Massachusetts, U.S.A.) site of the CRISPR/Cas9 system vector PX330 (Addgene, Cambridge, Massachusetts, U.S.A.). The plasmids used as the standard templates for quantitative RT-PCR were constructed by PCR amplification of the UL30, RS1, and RS1 promoter fragments, followed by ligation of the fragments into the pGM-T vector (Tiangen, Beijing, China). Plasmids expressing UL7 or UL7-MU were constructed by PCR amplification of the desired coding sequence, followed by ligation of the fragments into pcDNA3. The UL7 and UL7-MU coding sequences were amplified using the primers UL7-F and UL7-R, cut with EcoRI and XhoI restriction enzymes (TAKARA, Dalian, Liaoning, China) and ligated into the EcoRI and XhoI sites of pcDNA3. The plasmids were transfected into 293 T or Vero cells using the Fugene HD (Promega, Madison, Wisconsin, U.S.A.) reagent according to the manufacturer’s protocol. The specific primer sets used are listed in Additional file 1: Table S2.

### Construction of recombinant mutant viruses

Based on the methods of Bi et al. [23], the recombinant virus with a partial UL7 deletion mutation was constructed via co-transfection of plasmids PX330-UL7-1 and PX330-UL7-2. Virus was harvested from infected 293 T cells at 48 h post-infection (p.i.), and viral genomic DNA was extracted using the TIANamp Virus RNA/DNA Kit (Tiangen, Beijing, China). The genomic region surrounding the CRISPR target site of the UL7 gene was PCR-amplified using PrimeSTAR DNA polymerase (TAKARA, Dalian, Liaoning, China) with the primers UL7-sense and UL7-antisense. The PCR products were purified using the Universal DNA Purification Kit (Tiangen, Beijing, China). Purified PCR products (400 ng) amplified from the genomic DNA extraction were re-annealed and treated with SURVEYOR nuclease (Transgenomics, Omaha, NE, U.S.A.). A nuclease capable of recognizing and digesting the previously mismatched nucleotides in the genome was used to confirm that the mutation in the UL7 gene was created by the CRISPR approach. The products were analyzed on 10 % TBE polyacrylamide gels, which were stained with ethidium bromide (EB) and imaged using a Bio-Rad Gel Doc gel imaging system. Quantification was based on the relative band intensities, as described by Cong et al. [24]. After detecting the mutation efficiency, mutated viruses were purified via plaque assay.

### Preliminary analysis of the UL7-MU and MU-complemented virus

Vero cells were transfected with a eukaryotic expression vector containing the UL7 gene (pcDNA3-UL7). After confirming UL7 expression, the cells were infected with the UL7-MU or WT strain and harvested for a subsequent round of infection (MU-complemented P1 or WT-control P1) after the cytopathic effect (CPE) was observed. The second generation of this complemented viral strain was harvested as MU-complemented P2 or WT-control P2. For the plaque assay, 10-fold serial dilutions from 10^-2^ to 10^-6^ of the virus material were made in DMEM without FBS. The HSV-1 wild-type (WT), UL7-MU, MU-complemented P1, MU-complemented P2, WT-control P1 and WT-control P2 virus dilutions were grown in Vero cells overlaid with 0.6 % agarose in growth medium at 34 °C or 37 °C. Three (37 °C) or five (34 °C) days p.i., the cells were fixed and stained with 0.2 % crystal violet in 20 % ethanol. The plaque morphology was imaged. HSV-1 WT, UL7-MU, MU-complemented P1 and MU-complemented P2 viruses were used to infect Vero cells at a multiplicity of infection (MOI) of 1 at 34 °C or 37 °C. The total virus yielded from the cell culture supernatants and the infected cells was harvested at several time points (h p.i.: 8, 16, 24, 32, 40, and 48). The titers of all samples were determined by standard virus titration on blank Vero cells.

### Experimental infection in mice

For the acute infection assay, BALB/c mice were lightly anesthetized with ether. Following anesthesia, the BALB/c mice were infected via intranasal instillation of 2.3*10^5^ plaque-forming units (PFU) of HSV-1 virus (WT, UL7-MU or MU-complemented P1) or phosphate-buffered saline (PBS, sterile, pH 7.4) as a control. Tissues were obtained every one or two days p.i. followed by assessments of viral load and mouse organ pathology. The survival rate/mortality was assessed over a ten-day period. For the latency model, 4 BALB/c mice at 2 weeks of age were injected with either WT or UL7-MU virus through their foot pads at a dose of 5 x 10^3^/10 μl per mouse, followed by 3 months of observation. q-RT-PCR detection using specific primers for latency-associated transcript (LAT) mRNA in various organs of sacrificed mice was performed at 3 months after infection.

### Histopathologic and immunohistochemical examinations

Mouse organs were fixed in 10 % formalin and embedded in paraffin into tissue blocks. Paraffin-embedded samples were serially sectioned, generating approximately 5 5-μm sections/organ. Approximately 2 slides of each organ were stained with hematoxylin and eosin (H&E) to assess morphology. For immunohistochemical examinations, the specimens were immersed in 0.3 % hydrogen peroxide in PBS for 10 min to block intrinsic peroxidase activity. After three rinses with distilled water, the specimens were heated at 90 °C for 30 min for antigen retrieval [25]. After three rinses with PBS, the specimens were blocked with 5 % bovine serum albumin (BSA) in PBS at 37 °C for 15 min on a level surface in a humid chamber. The specimens were then incubated with a rabbit polyclonal anti-HSV-1 antibody (Abcam, Cambridge, U.K.) at 4 °C for 12 h. The sections were incubated with a mixture of secondary antibodies at 37 °C for 30 min after rinsing with PBS. Enzyme immunohistochemistry was performed using a standard avidin-biotin-peroxidase method with 3, 3’-diaminobenzidine (DAB) substrate (Tiangen, Beijing, China) according to the manufacturer’s protocol. After being rinsed with water for 6 min, the sections were stained with hematoxylin to assess cell nuclei. For each staining condition, a slide with rabbit IgG (Abcam, Cambridge, U.K.) was stained and examined in parallel as a control.

### Dual luciferase reporter assays

The Dual Luciferase Reporter Gene Assay Kit (Beyotime, Shanghai, China) was used to measure promoter activity. HEK293T cells were transfected with a reporter construct, pGL-α4, pGL-UL23 or pGL-UL41 [26] a UL7-WT or UL7-MU expression plasmid and the pRL-CMV plasmid, which expresses Renilla luciferase, as an internal control to normalize the transfection efficiency. All transfections were balanced for a total equal amount of DNA with the empty plasmid pcDNA3. Thirty-six hours after transfection, the cells were harvested, and the luciferase activities were measured using a luminometer. Each experiment was performed in triplicate, and the data represent the means ± standard deviations (SDs) of three independent experiments after normalization to Renilla activity.

### Quantitative RT-PCR

The viral load in mice was determined by qPCR with absolute quantitation. Based on the methods of Kessler et al. [27], the primers for the reaction were selected within a highly conserved region of the DNA polymerase gene UL30 from the HSV-1 genome, allowing for amplification of a 92-bp fragment from HSV-1 DNA. Simultaneously, a standard curve was produced from standard DNA samples (pGMT plasmid ligated with UL30 gene fragment). Viral genomic DNA was extracted from mouse tissue using a Universal DNA Purification Kit (Tiangen, Beijing, China). The TaqMan probe (Sangon Biotech, Shanghai, China) was labeled with 6FAM at the 5’ end and with TAMRA at the 3’ end. The reactions were performed using Premix Ex Taq TM (Probe qPCR) (Takara, Dalian, Liaoning, China) on an ABI 7500 (Life Technologies, Carlsbad, CA, U.S.A.).

For the relative quantification of LAT expression in mice, gene expression was calculated by relative quantitation using the comparative Ct method (ΔΔCt) using the mouse housekeeping gene GAPDH. Gene expression was expressed as the fold change (2^-ΔΔCt^) relative to samples from PBS-injected mice used as calibrators. Total RNA was extracted from mouse tissues using TRIzol reagent (Life Technologies, Carlsbad, CA, U.S.A.), and amplification reactions were performed using a One Step SYBR Prime Script™ PLUS RT-PCR Kit (Takara, Dalian, Liaoning, China).

The α-4 mRNA levels in Vero cells infected with UL7-MU or WT virus were also evaluated by absolute quantitation. A standard curve was produced from a standard RNA sample transcribed in vitro using T7 RNA polymerase. The template for transcription was a pGM-T plasmid ligated with the α-4 gene fragment. Total RNA was extracted using TRIzol reagent, and the amplification reactions were performed using a One Step SYBR Prime Script™ PLUS RT-PCR Kit.

The α-4 promoter DNA levels were also evaluated by CHIP assay with absolute quantitation. A standard curve was produced from a standard DNA sample (a pGM-T plasmid ligated with the α-4 gene promoter fragment). The amplification reactions were performed using a SYBR Premix Ex Taq II (Tli RNaseH Plus) Kit (Takara, Dalian, Liaoning, China). Template-negative and RT-negative reactions served as controls. The specific primer sets used are listed in Additional file 1: Table S2.

### Immunoblotting

Briefly, Vero cells in 60-mm dishes were transfected with pcDNA3-UL7 plasmid. At 24 h post-transfection, the cells were washed with PBS, and the cells were lysed with radioimmunoprecipitation assay (RIPA) lysis buffer. The obtained supernatants were subjected to SDS-PAGE, and the proteins were then transferred to polyvinylidene fluoride (PVDF) membranes. Membranes bearing proteins of interest were blocked in 5 % nonfat milk plus 0.1 % Tween 20 for at least 2 h. The membranes were probed with mouse polyclonal anti-UL7 (1:500) (Laboratory owned), followed by alkaline phosphatase-conjugated secondary antibody.

### Chromatin immunoprecipitation (ChIP)

The ChIP procedure and quantification were performed as previously described, with minor modifications [28]. Briefly, Vero cells were infected with the HSV-1 virus at an MOI of 10. At the indicated time points (h p.i.: 3, 4, 5, and 6), the cells were treated with 1 % formaldehyde for 10 min on ice to crosslink the proteins to DNA and quenched with 125 mM glycine. The cells were then washed with cold PBS and collected in 1 ml of cold PBS. The cell nuclei were extracted using a cell nucleus extraction kit (Sorlabio, Beijing, China) according to the manufacturer’s protocol. The nuclei were lysed in 1 ml RIPA lysis buffer for 1 h. The lysates were sonicated (20 rounds of sonication, 10 s each) to shear the DNA to between 200 and 1000 bp. The lysates were centrifuged at 12,000 g at 4 °C for 10 min and precleared with 30 μl of control agarose beads. After centrifugation at 2000 rpm at 4 °C for 3 min, the cell lysate supernatants were incubated overnight with UL7 antibody or negative-control IgG at 4 °C with rotation. Pretreated protein A + G beads (50 μl) were added, and the samples were incubated at 4 °C for an additional 2 h and then washed five times and eluted with 500 μl of Tris-EDTA buffer containing 1 % SDS. The elution solutions were reversed with 0.2 M NaCl at 65 °C overnight. The products were digested with RNase at 37 °C for 1 h and proteinase K at 45 °C for 2 h. The DNA was then extracted and analyzed using real-time PCR. The specific primer sets used are listed in Additional file 1: Table S3.

## Results

### Identification of the mutated UL7 gene in the viral strain

**Fig. 1:**
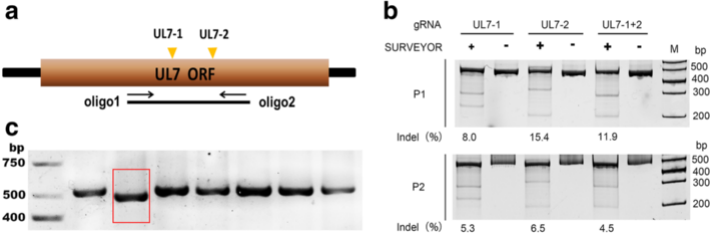
Identification of the mutated UL7 gene in the viral strain. **a** Design of the g-RNA sequences for UL7 gene mutation, with the target sites of the g-RNAs (UL7-1 and UL7-2) labeled in yellow. The fragments amplified by oligo 1 and oligo 2 were used in the SURVEYOR assay. **b** SURVEYOR detection of the mutated genes. Top: SURVEYOR assay of HSV-1 genomic DNA extracted from HEK293T cells expressing UL7-1 and UL7-2 individually or together infected with HSV-1 (P1); bottom: SURVEYOR assay of HSV-1 genomic DNA extracted from HEK293T cells expressing UL7-1 and UL7-2 individually or together infected with HSV-1 progeny virus (P2). **c** Identification of the mutated UL7 gene in the viral strain. The UL7 mutant was identified via PCR using UL7-sense and UL7-antisense primers. The mutated UL7 gene is indicated with a red box

Prior to the structural analysis of the UL7 gene sequence in the HSV-1 genome, we designed 2 g-RNA sequences to use in the CRISPR/Cas9 system (Fig. 1a). The studies were performed in 3 experimental groups: plasmids PX330-UL7-1 and PX330-UL7-2, containing target sequences 1 and 2, were transfected individually into cultured 293 T cells as groups 1 and 2, respectively, with both plasmids containing sequences 1 and 2 transfected together into the cells as group 3, and the empty plasmid group serving as the control group. SURVEYOR detection of the mutant strain from the transfected cells as described in the methods indicated that there were approximately 8 % and 15.4 % mutations, respectively, in the UL7 gene containing the UL7-1 and UL7-2 target sequences (Fig. 1b); the mutation efficiency was 11.9 % in the mutant strain from the transfected cells by UL7-1 and UL7-2 co-transfection (Fig. 1b). To ensure the stability of the mutant strains, the mutated strains produced from harvesting the cells transfected with the two plasmids together were further cloned via plaque assays for screening and purification; the genomes of the strains were subsequently amplified by PCR for sequencing. Sequencing revealed a UL7-MU strain with a 30-bp deletion corresponding to amino acids 75–85 in the UL7 protein (Fig. 1c). These amino acids are located in the β-barrel structure of this protein and may be related to its functional conformation (http://www.sbg.bio.ic.ac.uk/~phyre/index.cgi). The sequence of UL7-MU is shown in Additional file 1.

### Biological characterization of the viral strain with the mutated UL7 gene

**Fig. 2:**
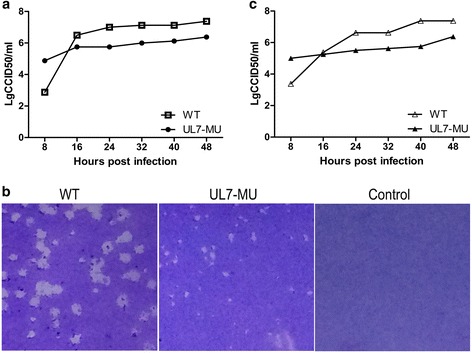
The mutated UL7 gene modulates viral proliferation. **a** Dynamic profile of the UL7-MU viral strain at 37 °C. Vero cells were infected with UL7-MU (filled circles) or HSV-1 WT (open boxes) at an MOI of 1. The supernatants and cells were harvested at the indicated time points, and the lysates were titered on Vero cells. **b** Morphological features of UL7-MU mutant plaques at 37 °C. Vero cells were infected with UL7-MU or HSV-1 WT at an MOI of 0.00001. The cells were fixed and stained 3 d p.i. **c** Dynamic profile of the UL7-MU viral strain at 34 °C. Vero cells were infected with UL7-MU (filled triangles) or HSV-1 WT (open triangles) at an MOI of 1. The supernatants and cells were harvested at the indicated time points, and the lysates were titered on Vero cells

In the identification of the mutant UL7-MU viral strain, the growth curve of UL7-MU on Vero cells was compared with that of the control WT viral strain. The growth tendency of the UL7-MU strain in cells at 37 °C and an MOI of 1 showed a clear decreasing at different time points (Fig. 2a), and the growth efficiency of the UL7-MU strain was approximately 10-fold lower than that of the WT strain (Fig. 2a). As shown in the plaque assay, there was a distinct difference in the plaque morphologies of the UL7-MU strain and the control strain grown on monolayers in Vero cells (Fig. 2b), and the extremely small diameters of the mutant plaques were consistent with that strain’s lower growth rate (Fig. 2b). These results suggest that the UL7 molecule is involved in HSV-1 viral replication. To determine if the mutation of UL7 leads to an alteration of the viral phenotype, the growth capacity of the mutant strain was compared with that of the WT under a different temperature environment (34 °C). Consistent with our prediction, there was a distinct difference between the plaque morphologies of the UL7-MU and WT viral strains grown on monolayers in Vero cells (data not shown). The growth curve of the UL7-MU strain at 34 °C and at the same MOI and time points was reduced by approximately 10-fold compared with that of the WT strain (Fig. 2c). Continuous observation of more than 15 passages of this mutated strain indicated that this altered phenotype was stable (data not shown). These data are sufficient to suggest that UL7 does play a supportive role in viral replication via an unknown mechanism.

### The mutant phenotype of UL7-MU is complemented by expressing the UL7 protein in cells

**Fig. 3:**
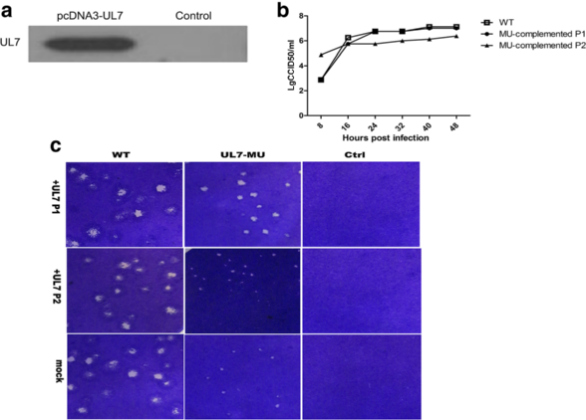
The mutated phenotype of UL7-MU is complemented by expression of the UL7 gene in cells. **a** The lysates of Vero cells transfected with the pcDNA3-UL7 plasmid were separated on a 12 % polyacrylamide gel, transferred to a PVDF membrane, and immunoblotted with UL7 antibody. The position of the UL7 protein is indicated. **b** Morphological features of plaques formed by the complemented UL7-MU and WT viral strains. Cells were infected at 24 h post-transfection with UL7-MU or WT virus at an MOI of 1. The supernatants and cells of the complemented virus were harvested after CPE was observed, followed by plaque assays. Vero cells were infected with MU-complemented P1, WT-control P1, MU-complemented P2, WT-control P2 or HSV-1 WT at an MOI of 0.00001. The cells were fixed and stained 3 d p.i. **c** Dynamic profiles of the MU-complemented P1 and MU-complemented P2 viral strains at 37 °C. Vero cells were infected with the MU-complemented P1 (filled circles), MU-complemented P2 (filled triangles) or HSV-1 WT (open boxes) at an MOI of 1. The supernatants and cells were harvested at the indicated time points, and the lysates were titered on Vero cells

Previous reports suggested that an HSV-1 strain with a mutated UL7 gene displays a morphological characteristic of small plaques [17], which was confirmed by our current observations. To confirm the correlation between the phenotype and the UL7 gene mutation, a complementation experiment was performed in which Vero cells were transfected with a eukaryotic expression vector containing the UL7 gene (pcDNA3-UL7). The expression of the UL7 protein was first assessed by Western blotting using a specific antibody against the UL7 protein. After the identification of UL7 expression (Fig. 3a), the cells were infected with the UL7-MU strain and harvested for a subsequent round of infection after the CPE was observed. The WT strain was also used to infect the transfected cells and harvested from this cell as a control. Predictably, a typical plaque similar to that induced by the WT control was observed after the virus was harvested from the complementation experiment in transfected Vero cells (Fig. 3b), while a growth curve similar to that of the WT control was recorded from cells infected with the virus harvested from the complementation experiment (Fig. 3c). Interestingly, further testing of the second generation of this complemented viral strain compared with the WT control indicated the recovery of the small-plaque and lower-proliferation phenotypes of the mutated strain in cells (Fig. 3b and c). These results verified the correlation between the UL7 molecule and the mutant phenotype, at least in cultured cells.

### A mutated HSV-1 UL7 gene attenuates virulence in infected mice

**Fig. 4:**
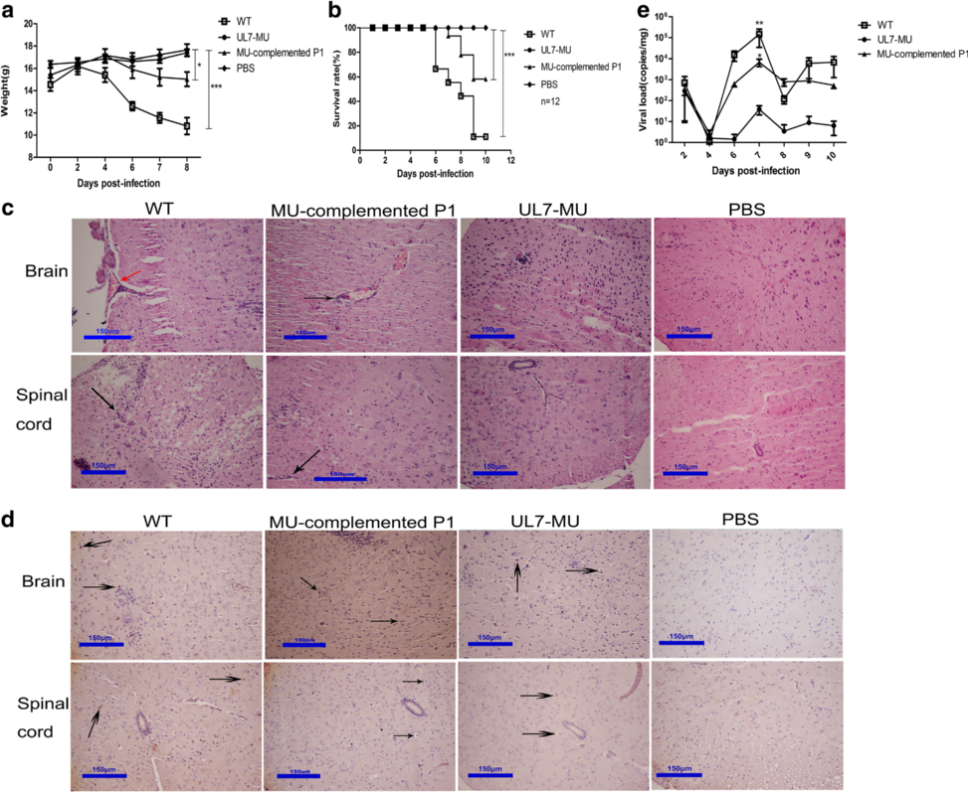
The UL7-MU viral strain exhibits lower virulence in infected mice compared with the WT strain. **a** BALB/c mice were infected via intranasal instillation of 2.3 × 10^5^ PFU of WT HSV-1 (open boxes), UL7-MU (filled circles), MU-complemented P1 (filled triangles) or PBS (open rhombuses) as a control. The weights of the mice were measured every 2 days. Data are shown as means ± SD. ^∗^ *P* < 0.05; ^∗∗∗^ *P* < 0.005. **b** The survival rates of mice infected with WT HSV-1 (open boxes), UL7-MU (filled circles), MU-complemented P1 (filled triangles) or PBS (open rhombuses). **c** Pathological changes in the CNS tissues and spinal cords of mice infected with WT, UL7-MU, MU-complemented P1 or PBS. Tissue sections were stained with H&E and imaged using an optical microscope at 200× magnification. Tissue hyperemia is highlighted with red arrows; infiltration of inflammatory cells is highlighted with black arrows. **d** HSV-1 immunohistochemistry of CNS tissues and spinal cords in WT, UL7-MU, MU-complemented P1 or PBS-infected mice. Non-specific staining was observed in PBS-infected mice. Positive expression of the HSV 1 antigen was detected in WT, UL7-MU and MU-complemented P1 infected mouse CNS tissues and spinal cords (arrows). **e** Viral load detection in the CNS of mice challenged with WT (open boxes), UL7-MU (filled circles) or MU-complemented P1 (filled triangles) viruses, as determined by real-time RT-PCR. Viral copy numbers were quantified according to an HSV-1 DNA standard. Data are shown as the means ± SD (three independent mice). ^∗^ *P* < 0.05; ^∗∗^ *P* < 0.01

Given that the mutant HSV-1 UL7 gene led to decreased viral proliferation capacity, we further characterized this strain’s virulence in infected mice. Prior work has shown that mice can be used as an animal model for acute HSV-1 infection to effectively address pathologic injury induced by viral infection [29–31]. As evidenced by clinical observations of BALB/c mice infected with the WT, UL7-MU or MU- complemented P1 strains (infection dosage: 2.3x10^5^ PFU/50 μl) via the nasal route, the UL7-MU strain exhibited reduced virulence, as evidenced by slight, abnormal changes in the mice, in contrast to the arched back and down hair, markedly lower activity levels and worse status in mice infected with WT or MU-complemented P1 virus. Furthermore, the weight variation profiles indicated that the weights of mice infected with the WT or MU-complemented P1 strain clearly decreased, whereas the weights of mice infected with the UL7-MU viral strain tended to increase regardless of the slight differences identified in the negative control group (Fig. 4a). Importantly, the death rates of the mice infected with the WT, MU-complemented P1 or UL7-MU viral strains were 91.7 %, 41.7 % and 0 %, respectively, within the 10-day observation period (Fig. 4b); the longest survival time of the mice infected with the UL7-MU strain was over 4 months. Further pathological examinations of the mice in the 3 groups sacrificed at 4, 6, 7, 8, 10 days p.i. indicated distinct differences in the central nervous system (CNS), characterized by clear inflammatory features in the cerebral parenchyma and nervous meninges, such as perivascular infiltration of inflammatory cells, tissue hyperemia and inflammatory cell aggregation (Fig. 4c), as well as local vacuolated alteration and inflammatory cell infiltration in spinal tissues of mice infected with the WT and MU-complemented P1 strains (Fig. 4c). Such pathological changes tended to increase in severity with time. However, only a small number of inflamed cells, associated with slight perivascular infiltration and tissue damage, were noted in the CNS of mice infected with the UL7-MU viral strain (Fig. 4c). The neurovirulence scoring of the pathological changes in the CNS tissues of the mice in the 3 groups indicated significantly higher neurovirulence in mice infected with the WT and MU-complemented P1 viruses compared with mice infected with the UL7-MU viral strain (Table 1); there were significant differences in the pathological examinations of sections of 3 CNS or spinal cord tissues each from 30 mice in the 3 groups (10 in each group) (Table 1). Parallel immunohistochemistry assays using an anti-HSV-1 antibody showed a basic correlation between the aforementioned pathological changes and viral antigen expression (Fig. 4d). Further quantitative viral genomic DNA detection by PCR for the CNS tissues of mice sacrificed at different time points in the 3 groups revealed that viral load increases in the tissues were closely linked to the pathological changes in the CNS tissues of mice infected with the WT or MU-complemented P1 strains (Fig. 4e); in contrast, no remarkable proliferation was noted in the CNS tissues of mice infected with the UL7-MU viral strain, and the viral load was much lower than that in mice infected with the WT or MU-complemented P1 strains (Fig. 4e).

**Table 1:** Neurovirulence of mice infected with 2.3 × 10^5^ PFU/50 μl HSV-1 wild type, MU-complemented P1 and UL7-MU strain

Days	Groups							
	Wild-type		MU-complemented P1		UL7-MU		Negative control	
	CNS	Spinal cord	CNS	Spinal cord	CNS	Spinal cord	CNS	Spinal cord
4	+	+	±	±	±	±	-	-
6	+	+	+	+	±	±	-	-
7	+	+	+	+	±	±	-	-
8	+	+	+	+	±	±	-	-
10	+	++	+	+	±	±	-	-

Scale as follows

Grade -, normal tissue

Grade+, slight infiltration of inflammatory cells without neural damage

Grade++, slight neural damage with inflammatory cell infiltration

Grade+++, massive neural damage with inflammatory cell infiltration

Grade++++, serious neural damage with inflammatory cell infiltration

### Attenuated phenotype of the mutated strain is observed in latent infection

**Fig. 5:**
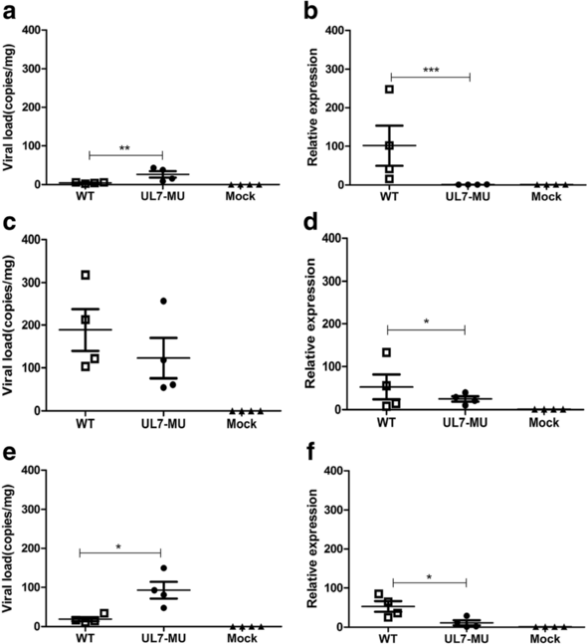
The UL7-MU viral strain exhibits attenuated phenotypes in a latent mouse infection model compared with the WT strain. **a** BALB/c mice were infected with WT HSV-1, UL7-MU or PBS via the foot pad at a dose of 5x10^3^/10 μl per mouse. The viral load was detected in the CNS of mice challenged with WT (open boxes), UL7-MU (filled circles) or PBS (filled triangles) by absolute real-time RT-PCR. Viral copy numbers were quantified according to the HSV-1 DNA standard pGM-T UL30 plasmid. **b** The levels of LAT expression in the CNS of mice challenged with WT (open boxes), UL7-MU (filled circles) or PBS (filled triangles), as determined by relative real-time RT-PCR. The graphic indicates the fold change of RNA levels in virus-infected mice compared to PBS-injected mice. The mouse housekeeping gene GAPDH was used to normalize quantities in mouse tissue. Relative quantification was performed by the comparative Ct method (△△Ct) using RNA from PBS mice as a calibrator. **c** Viral load detection in the spinal cord of mice challenged with WT (open boxes), UL7-MU (filled circles) or PBS (filled triangles). **d**The levels of LAT expression in the spinal cord of mice challenged with WT (open boxes), UL7-MU (filled circles) or PBS (filled triangles). **e** Viral load detection in the trigeminal nerve of mice challenged with WT (open boxes), UL7-MU (filled circles) or PBS (filled triangles). **f** The levels of LAT expression in the trigeminal nerves of mice challenged with WT (open boxes), UL7-MU (filled circles) or PBS (filled triangles). Data are shown as the means ± SD (experiments done once in triplicate). ^∗∗∗^ *P* < 0.005; ^∗∗^ *P* < 0.01; ^∗^ *P* < 0.05

The most important characteristic of HSV-1 infection in humans is the ability of the virus to induce a latent infection. Fortunately, this mode of infection can also be studied in mice by injecting the virus into the foot pad [32]. Using this model, the phenotype of the mutated strain in latent viral infection was investigated further, in which 4 BALB/c mice at 2 weeks of age were injected with the WT or UL7-MU virus through their foot pads at a dose of 5 x 10^3^/10 μl per mouse, followed by 3 months of observation. Viral RNA and DNA were extracted from various organs from mice sacrificed at 3 months after infection. Quantitative PCR detection of viral genomic DNA in the CNS and trigeminal nerve tissues of mice infected by WT revealed a slightly lower number of copies compared to mice infected by UL7-MU (Fig. 5a, e), but a slightly higher number of copies of genomic DNA in was observed in the spinal cord in mice infected by WT than in mice infected by UL7-MU (Fig. 5c). However, q-RT-PCR detection using primers specific for the LAT mRNA performed in the CNS, spinal cord and trigeminal nerve of mice sacrificed at 3 months after infection suggested that the expression of LAT was significantly lower in the CNS of UL7-MU-infected mice than in mice infected with the WT virus (Fig. 5b); moreover, LAT mRNA expression levels in the spinal cord and trigeminal nerve were approximately 2-fold lower in UL7-MU-infected mice than in mice infected with the WT strain (Fig. 5d and f). These seemingly contradictory observations suggest that first, UL7-MU is capable of entering neurons during latent infection, and second, the mutated UL7 does block transcription of LAT mRNA from the viral genome in neurons to some extent. However, because UL7-MU is an attenuated strain, it might be reasonable to speculate that the higher number of copies of viral DNA of UL7-MU in some neurons despite the very low LAT transcription of this viral strain is due to the lower immune response of the host to the virus.

### UL7 protein is involved in regulating the transcriptional activation of the HSV-1 α-4 gene

**Fig. 6:**
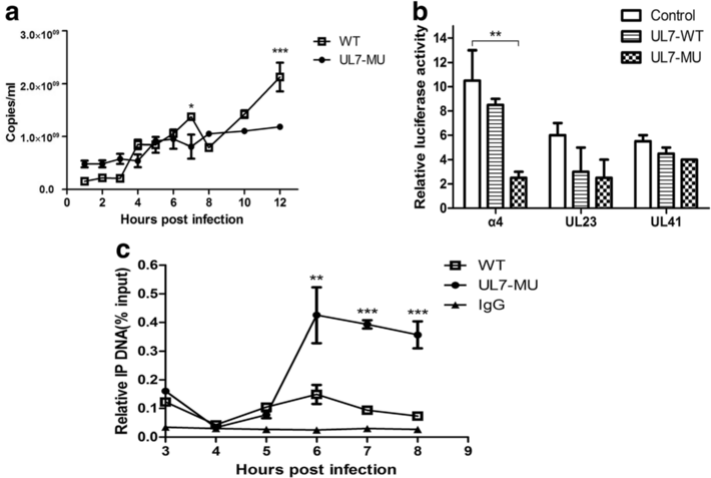
UL7 protein is involved in regulating the transcriptional activation of the HSV-1 α-4 gene. **a** Comparison of α-4 transcriptional efficiencies during the proliferetion of UL7-MU (filled circles) and WT viral strains (open boxes). Gene expression levels were detected using absolute real-time RT-PCR. Gene copy numbers were quantified according to the gene RNA standard. **b** HEK293T cells were co-transfected with pGL-α-4, pGL-UL23 or pGL-UL41 and UL7-WT, UL7-MU or control plasmid for 36 h before luciferase activities were quantified. Data are shown as means ± SD. ^∗∗^ *P* < 0.01. Each group was repeated at least three times. **c** Vero cells were infected with HSV-1 WT (open boxes) or UL7-MU (filled circles) for 3, 4, 5 and 6 h followed by ChIP assay. The α-4 promoter level was determined using absolute real-time RT-PCR. Gene copy numbers were quantified according to the gene DNA standard. The error bars indicate the standard deviation (SD). Data are shown as the means ± SD (three independent experiments). ^∗∗^ *P* < 0.01

To investigate the role of the UL7 protein during viral infection, we first compared the transcriptional efficiencies of the α-4 gene in the immediate-early phase of the WT strain versus the UL7-MU strain. As shown by q-RT-PCR amplification of α-4 gene of UL7-MU and WT strains at different time points, the transcription levels of this gene in UL7-MU-infected cells were reduced beginning at 7 h p.i. compared with the clear peak in the transcriptional level identified in WT strain-infected Vero cells (Fig. 6a). In further experiments, eukaryotic expression plasmids encoding either recombinant WT or mutated UL7 genes (pcDNA3-UL7-MU, pcDNA3-UL7-WT) were co-transfected with a dual-luciferase reporter plasmid containing the recombinant HSV-1 α-4, UL23 or UL41 gene promoters into HEK293T cells to measure the luminescence intensity of the harvested cells at 36 h according to the standard protocol; the transcriptional efficiencies were then determined. The mutated UL7 gene exhibited a remarkable inhibitory effect on the transcriptional efficiency of the α-4 gene compared with the WT UL7 gene but not on the UL23 and UL41 genes (Fig. 6b). Based on these results, we speculate that the UL7 protein plays a modulatory role in viral α-4 gene transcriptional activation or regulation. To confirm this hypothesis, we further performed ChIP assays to produce the immunoprecipitated complex from Vero cell lysates infected with either the WT or UL7-MU strain at 3, 4, 5, and 6 h p.i. using an anti-UL7 specific antibody. Subsequent q-PCR amplification of the complex using viral α-4 gene promoter-specific primers was performed to observe the involvement of UL7 in gene transcription. The results indicated that only the number of α-4 gene copies in the complex precipitated from cell lysates infected with the WT strain tended not to change over time (Fig. 6c). In contrast, the number of gene copies increased over time in the precipitated complex in cells infected with the mutant strain (Fig. 6c). This finding suggests that the binding of the transcriptional complex with mutated UL7 to the α-4 gene promoter motif did not immediately initiate transcription of the viral genome, whereas the transcriptional complex with WT UL7 performed its function on the viral genome to lead to the production of mRNA of immediate-early proteins for viral replication. These data suggest that the UL7 protein is involved in the transcriptional activation or regulation of the α-4 gene.

## Discussion

Based on previous reports, more than 20 tegument proteins, which comprise the most important HSV-1 viral structures, are frequently identified as localizing to the region between the viral nucleocapsid and the extracellular membrane [33]. These molecules exhibit distinct molecular biological functions and play major roles in individual events of viral infection and subsequent proliferation. The current data substantially demonstrated that these tegument proteins play essential roles in viral entry into the nucleus [34], nucleocapsid transfer [35–37], viral gene transcriptional activation and co-regulation [38, 39], viral/cell gene expression [40], viral gene replication [41] and other biological events. These molecules are present at different ratios in the virion structure depending upon their functional requirements in viral replication. The fraction of UL7 with respect to total tegument protein molecules is known to be only 1 %, suggesting a potential role as a regulatory molecule.

In our study, a mutant viral strain containing a 30-bp deletion in the nucleotide 225–254 region of the UL7 gene, which encodes amino acids 75–85 in the β-barrel structure of this protein, was constructed using the CRISPR/Cas9 system to investigate the potential functional significance of the UL7 tegument protein in viral proliferation. Compared with the WT strain, the biological phenotype of the UL7-MU strain was characterized by lower proliferative efficiency and altered plaque morphology. These phenotypic characteristics appear to suggest an important role of the UL7 molecule in viral infection and proliferation, confirming the description of the lower growth phenotype of the UL7 mutant strain by Tanaka et al. This modified phenotype was not specifically impacted by environmental temperatures, whereas the morphological features of the plaques suggested lower viral infectivity. Further observation after passages of the mutant indicated that the altered phenotype was steadily maintained for at least more than 15 passages. These results confirmed the correlation of the weaker virulence phenotype with the deletion of amino acids 75–85. Nasal infection of mice with the UL7-MU strain resulted in very slight clinical manifestations and slight inflammatory symptoms in the CNS, which were associated with a low level of viral proliferation in CNS tissues. Long-term survival was observed for the mice infected with the UL7-MU strain. In contrast, infection with the WT strain led directly to mouse death within 10 d p.i. Interestingly, a decrease in LAT mRNA expression was observed in the CNS and trigeminal nerve of mice infected with UL7-MU via the foot pad at 3 months p.i., suggesting dramatically decreased transcription of the viral genome of the mutant in the neurons. These phenotypic characteristics, presumably triggered by the mutation, suggest that the UL7 tegument protein plays important regulatory roles in viral infection and proliferation. Based on these results, we then analyzed a variety of gene transcription events induced by infection with the UL7-MU and WT strains at the same MOI. We observed an approximately 2-fold decrease in the transcriptional levels of the immediate-early gene α-4, which was noted as the major biological event during infection with this mutant strain. This result was again confirmed by quantitative detection of the levels of transcription of the α-4 gene after co-transfection of the mutant UL7 gene into HEK293T cells with a dual-luciferase reporter plasmid; by contrast, the transcription of the E and L genes was not influenced by the mutant UL7 gene. These observations limit the role UL7 plays in viral infection to that of a viral α-4 gene transcriptional activator or regulator.

Previous studies of the mechanism of HSV-1 infection have demonstrated that the critical event of viral replication in cells is the transcriptional activation of the α-4 gene, which is triggered by the interaction of the classic VP16/Oct1/HCF1 complex and the promoter motif of α-4 gene associated with some undetermined cellular and viral molecules [42, 43]. Based on the observations obtained in this work, the virally encoded molecules directly involved in this process to ensure the mechanism of α-4 gene transcriptional activation include, at least, the UL7 molecule. Our subsequent ChIP assays further revealed a marked increase in the production of the mutated UL7-α-4 gene complex during viral genome transcriptional activation in UL7-MU-infected cells compared with WT-infected cells, which suggested that the transcriptional complex with mutated UL7 stagnated at the α-4 gene promoter motif and retarded transcription of the viral genome. This result suggests that UL7 functions to assist in the transcriptional activation or regulation of the viral α-4 gene and that the mutated molecule is able to decrease this transcriptional efficacy. However, whether this molecule is involved in the interaction between the transcriptional complex and the promoter motif of the viral α-4 gene or is related to the process of viral genomic chromatin remodeling remains to be determined. Additional studies are needed to fully elucidate the mechanism of UL7 in this process.

## Conclusion

This analysis of UL7 protein function during HSV-1 infection in tissue culture and mice demonstrated for the first time that this viral protein plays an important role in the transcriptional activation or regulation of the HSV-1 α-4 gene during viral infection.

## Additional file

Additional file 1: Table S1. DNA sequences of gRNAs and primers used for SURVEYOR assay. **Table S2**. Primers used for plasmid construction and qPCR. **Table S3**. Primers used for qPCR of CHIP assay. The DNA sequence of mutated UL7 gene of HSV-1 strain. (DOCX 16 kb)

## Abbreviations

CHIP: chromatin immunoprecipitation
CNS: central nervous system
CPE: cytopathic effect
H&E: hematoxylin and eosin
HSV-1: Herpes Simplex Virus type I
LAT: latency-associated transcript
PBS: phosphate-buffered saline
PVDF: polyvinylidene fluoride
RIPA: radioimmunoprecipitation assay
SD: standard deviation
WT: wild-type
