# Supplementary material for: The mutated tegument protein UL7 attenuates the virulence of herpes simplex virus 1 by reducing the modulation of α-4 gene transcription
Source: Virol J. 2016 Sep 13;13(1):152. doi: 10.1186/s12985-016-0600-9 (PMC5020468; doi:10.1186/s12985-016-0600-9)
Supplement: Additional file 1: Table S1. — DNA sequences of gRNAs and primers used for SURVEYOR assay. Table S2. Primers used for plasmid construction and qPCR. Table S3. Primers used for qPCR of CHIP assay. The DNA sequence of mutated UL7 gene of HSV-1 strain. (DOCX 16 kb) [file 12985_2016_600_MOESM1_ESM.docx]

**Supplementary materials**

**Table S1. DNA sequences of gRNAs and primers used for SURVEYOR assay**

| Primer name | Assay | Genomic target | Primer sequence (5' to 3') |
| --- | --- | --- | --- |
| UL7-1 top | PX330-UL7-1 construct | UL7 | caccggttgcggttcgttctggac |
| U7-1 bottom |  |  | aaacgtccagaacgaaccgcaacc |
| UL7-2 top | PX330-UL7-2 construct | UL7 | caccggacgcctatgtgacgtcgg |
| UL7-2 bottom |  |  | AAACCCGACGTCACATAGGCGTCC |
| UL7-sense | SURVEYOR assay | UL7 | AGATCGCGGTCGCCCGGAAGAACTG |
| UL7-antisense |  |  | AGTTCTCCAGGTACATCAGCAGACA |

**Table S2. Primers used for plasmid construction and qPCR**

| Primer name | Assay | Genomic target | Primer sequence (5' to 3') |
| --- | --- | --- | --- |
| UL7-F | pcDNA3 construct | UL7 | GGAATTCATGGCCGCCGCGACGGCCGACGAT |
| UL7-R |  |  | CCGCTCGAGTCAACAAAACTGATAAAACAGCG |
| UL30-F | Absolute quantification RT-PCR | UL30 | CATCACCGACCCGGAGAGGGAC |
| UL30-R |  |  | GGGCCAGGCGCTTGTTGGTGTA |
| TaqMan probe |  |  | 5’-6FAM-CCGCCGAACTGAGCAGACACCCGCGC-TAMRA |
| LAT-QF | Relative quantification RT-PCR | LAT | GCTGGTGTACCTGATAGTG |
| LAT-QR |  |  | GCCCGTCCAGATAAAGTC |
| GAPDH-QF | Relative quantification RT-PCR | GAPDH | GGTGAAGGTCGGTGTGAACG |
| GAPDH-QR |  |  | CTCGCTCCTGGAAGATGGTG |
| α-4-QF | Absolute quantification RT-PCR | α-4 | CTGCTGGCCTCCATGGTAGA |
| α-4-QR |  |  | TCATCGTCGTCGGCTCGAA |

Table S3. Primers used for qPCR of CHIP assay

| Primer name | Assay | Genomic target | Primer sequence (5' to 3') |
| --- | --- | --- | --- |
| α-4-2-QF | Absolute quantification RT-PCR | α-4 promoter | CAGAGACAGACCGTCAGAC |
| α-4-2-QR |  | α-4 promoter | CGATGCTTGGGTGGGAAA |

**File S1. The DNA sequence of mutated UL7 gene of HSV-1 strain**

ATGGCCGCCG CGACGGCCGA CGATGAGGGG TCGGCCGCCA CCATCCTCAA GCAGGCCATC GCCGGGGACC GCAGCCTGGT CGAGGCGGCC GAGGCGATTA GCCAGCAGAC GCTGCTCCGC CTGGCCTGCG AGGTGCGCCA GGTCGGCGAC CGCCAGCCGC GGTTTACCGC CACCAGCATC GCGCGCGTCG ACGTCGCGCC TGGGTGCCGG TTGCGGTTCG TTCTGTCGGA GGATTACTTT AAGCGCTGCT GCGGTCAGTC CAGTTATCGC GGCTTCGCGG TGGCGGTCCT GACGGCCAAC GAGGACCACG TGCACAGCCT GGCCGTGCCC CCCCTCGTTC TGCTGCACCG GTTCTCCCTG TTCAACCCCA GGGACCTCCT GGACTTTGAG CTTGCCTGTC TGCTGATGTA CCTGGAGAAC TGCCCCCGAA GCCACGCCAC CCCGTCGACC TTTGCCAAGG TTCTGGCGTG GCTCGGGGTC GCGGGTCGCC GCACGTCCCC ATTCGAACGC GTTCGCTGCC TTTTCCTCCG CAGTTGCCAC TGGGTCCTAA ACACACTCAT GTTCATGGTG TACGTAAAAC CGTTCGACGA CGAGTTCGTC CTGCCCCACT GGTACATGGC CCGGTACCTG CTGGCCAACA ACCCGCCCCC CGTTCTCTCG GCCCTGTTCT GTGCCACCCC GACGAGCTCC TCATTCCGGC TGCCGGGGCC GCCCCCCCGC TCCGACTGCG TGGCCTATAA CCCCGCCGGG ATCATGGGGA GCTGCTGGGC GTCGGAGGAG GTGCGCGCGC CTCTGGTCTA TTGGTGGCTT TCGGAGACCC CAAAACGACA GACGTCGTCG CTGTTTTATC AGTTTTGTTG A
